# Solvation Free Energies from Machine Learning Molecular Dynamics

**DOI:** 10.1021/acs.jctc.4c00116

**Published:** 2024-05-21

**Authors:** Nicéphore Bonnet, Nicola Marzari

**Affiliations:** Theory and Simulation of Materials, Ecole Polytechnique Fédérale de Lausanne, 1015 Lausanne, Switzerland

## Abstract

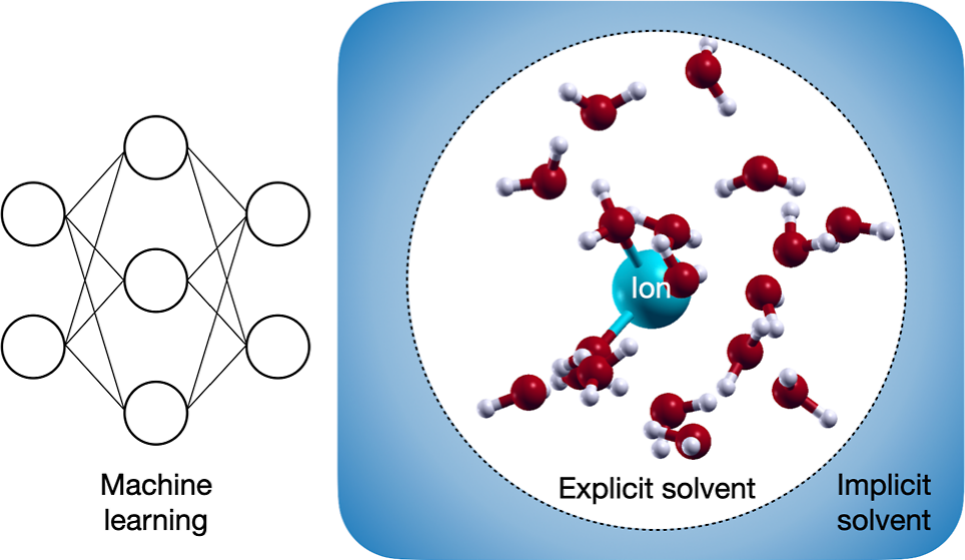

The present work proposes an extension to the approach of [Xi, C; et al. *J. Chem. Theory Comput.***2022,***18,* 6878] to calculate ion solvation free energies from first-principles (FP) molecular dynamics (MD) simulations of a hybrid solvation model. The approach is first re-expressed within the quasi-chemical theory of solvation. Then, to allow for longer simulation times than the original first-principles molecular dynamics approach and thus improve the convergence of statistical averages at a fraction of the original computational cost, a machine-learned (ML) energy function is trained on FP energies and forces and used in the MD simulations. The ML workflow and MD simulation times (≈200 ps) are adjusted to converge the predicted solvation energies within a chemical accuracy of 0.04 eV. The extension is successfully benchmarked on the same set of alkaline and alkaline-earth ions.

## Introduction

Solvation and electrolytic effects play a key role in electrochemical energy conversion devices and advanced water treatment and separation processes.^1−3^ While implicit solvent models have been often used in first-principles (FP) molecular dynamics (MD) simulations,^4,5^ they generally cannot capture specific molecular effects that may be important at the solute/solvent interface. Conversely, an explicit treatment of all solvent molecules is often computationally intractable. Alternatively, hybrid approaches involve treating a few inner solvation layers explicitly, and the rest of the solvent implicitly.^6,7^ Such approaches receive a rigorous statistical treatment within the quasi-chemical theory (QCT) framework of Pratt and co-workers,^8−10^ whereby solvation properties are expressed as a sum of free-energy contributions, each one involving a fixed number of solvent molecules within the inner solvation shell. In turn, the free-energy contributions can be calculated by various methods, including thermodynamic integration,^11^ and stochastic sampling and relaxation of solvation structures.^10,12,13^ However, those methods may remain challenging in terms of computational intensity or accuracy. Alternatively, in a recent work,^14^ Xi et al. have used an approach to directly calculate the free energy of the hybrid system from first-principles molecular dynamics (FPMD) simulations. Their predictions of alkaline (earth) ion solvation free energies were in good agreement with the experiment. However, their FP trajectories were limited to 15 ps, preventing them from converging the average total energy within less than 0.2–0.3 eV.

In the present work, after contextualizing this approach within the appropriate QCT framework, we introduce a machine learning extension to perform longer MD simulations and improve energy convergence. This extended approach is satisfactorily benchmarked for the same set of cations.

## Methodology

### General Approach

**Figure 1:**
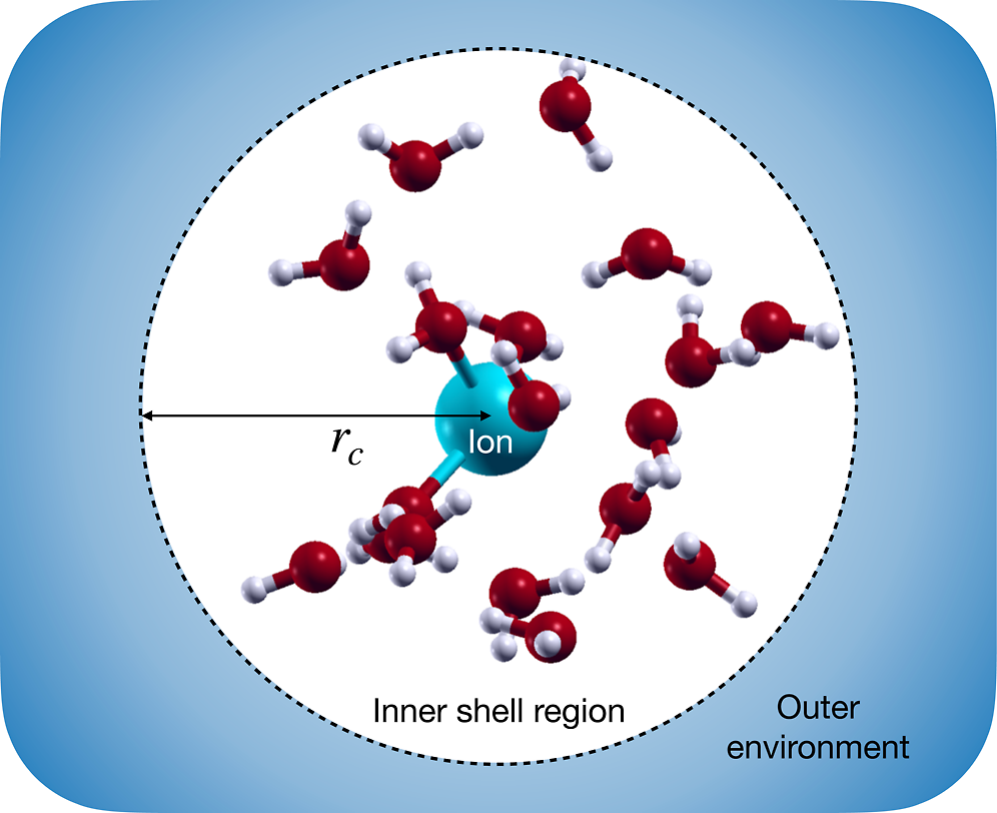
Hybrid solvation model comprising an explicit inner shell region and an implicit outer environment.

Given an ion in solution, the inner shell region is defined as the set of points within a distance *r*_c_ from the ion, and the outer environment as the rest of space (Figure 1). The probability to find exactly *n* solvent molecules in the inner shell at equilibrium is denoted by *P*_*i*_(*n*) when the ion interacts with the solvent, and by *P*_0_(*n*) when the interaction is fictitiously switched off (noninteracting ion). Within QCT, one shows that the ion solvation free energy can be expressed as^10^
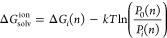
1where *k* is Boltzmann’s constant, *T* is the temperature, and Δ*G*_*i*_(*n*) is the solvation free energy with an inner shell constrained to contain exactly *n* solvent molecules. In practice, this formula is useful if the probability distributions *P*_*i*_(*n*) and *P*_0_(*n*) have sufficient overlap so that their ratio can be reliably estimated for some value of *n*. This is the case for the small solutes considered in the present study, while for larger solutes, other formulas are typically used.^10^

First, the probability ratio in eq 1 is addressed. In principle, *P*_*i*_(*n*) and *P*_0_(*n*) must be obtained at a fixed chemical potential of the solvent molecules, as achieved for instance via grand canonical Monte Carlo simulations.^15^ Here, instead, we find an estimate of the probability distributions by running MD simulations in the canonical ensemble. The ion is placed at the center of a cubic box filled with 57 water molecules, representing a concentration of ≈1 M, and the volume of the simulation box is adjusted to the absolute molar volume of the ion as given by ref (16). A FPMD simulation of 1 ps (equilibration) + 10 ps is carried out in periodic-boundary conditions (PBC) at 300 K. Then, for an inner shell strictly enclosed within the simulation box, the probabilities *P*_*i*_(*n*) and *P*_0_(*n*) are obtained by counting the corresponding configurations over the 10 ps sampling period. The validity of this approximation is discussed a posteriori (see Results section).

Next, the term Δ*G*_*i*_(*n*) is addressed. It is by definition obtained as
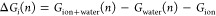
2where *G*_ion+water_(*n*) and *G*_water_(*n*) are, respectively, the free energies of the ion + water system and water system subject to the *n*-molecules inner shell constraint, and *G*_ion_ is the free energy of the ion in vacuum. Referring to the *n* water molecules of the inner shell as the water cluster, we further decompose the free energies into
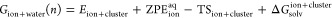
3
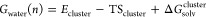
4where *E*_*X*_ and *S*_*X*_ are the enthalpy and entropy of the system *X* (ion + water cluster, or water cluster only) in vacuum, Δ*G*_solv_^*X*^ is its solvation free energy in the outer environment, and ZPE_ion_^aq^ is the zero-point energy of the solvated ion. Likewise, the unsolvated ion free energy is obtained as *G*_ion_ = *E*_ion_ + ZPE_ion_^vac^ – TS_ion_, where *E*_ion_, ZPE_ion_^vac^ and *S*_ion_ are, respectively, its enthalpy, zero-point energy, and entropy.

In their work,^14^ Xi et al. use an inner water cluster of 18 molecules, shown to encompass the first and second solvation layers of the ion. They obtain *E*_*X*_ + Δ*G*_solv_^*X*^ as the average total energy ⟨*E*_tot_^*X*^⟩ of a FPMD simulation. The total energy *E*_tot_^*X*^ of each MD step is calculated as the DFT energy of the explicit system *X* embedded in a continuum dielectric medium, capturing the effect of the outer solvation environment. The continuum model of Andreussi et al.^5^ is used as implemented in the Environ package^17^ of Quantum ESPRESSO.^18^ In the MD simulation, a confining potential wall is applied around the water cluster to prevent its diffusion into the outer solvation environment. The radius of this spherical wall is dynamically adjusted to maintain the wall pressure around a preset value. The entropies *S*_*X*_ and zero-point energy ZPE_ion_^aq^ are calculated based on the vibrational spectra obtained from the force–force correlation functions in a quasi-harmonic approximation. Finally, the energy of the unsolvated ion is calculated by DFT, and for a monatomic ion, ZPE_ion_^vac^ = 0 and its entropy is obtained from the perfect gas translational partition function.

The present work follows the same general approach but with some notable differences as listed below:

- *E*_*X*_ alone is calculated as the average total energy ⟨*E*_tot_^*X*^⟩ of the MD simulation, while Δ*G*_solv_^*X*^ is treated separately (see second bullet). To allow for longer MD simulation times, the total energy *E*_tot_^*X*^ is obtained from a machine-learned (ML) potential energy of the explicit system (see details below).
- Instead of using the continuum dielectric medium, the effect of the outer environment is treated analytically. Δ*G*_solv_^*X*^ is taken as the sum Δ*G*_hyd_^*X*^ + Δ*G*_diel_^*X*^, where Δ*G*_hyd_^*X*^ is the free energy of hydration, originating from hydrogen bonds created around the cluster, and Δ*G*_diel_^*X*^ is the free energy change coming from dielectric screening by the outer solvation environment. Following Born’s equation (in atomic units),^19^

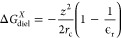
5where *z* is the electric charge of *X* and ϵ_r_ is the relative permittivity of the solvent in the bulk (here taken to be 78). As a decoupling hypothesis, Δ*G*_hyd_^*X*^ is assumed, at a given density, to depend only on the cluster size (and not on its charge). Then, for a given cluster radius,  and Δ*G*_hyd_^cluster^ cancel out in the expression of *G*_ion+water_(*n*) – *G*_water_(*n*).

- In line with the QCT approach, MD simulations are performed at a fixed radius *r*_c_ of the potential wall acting on the oxygen atoms of the water molecules. The value of *r*_c_ = 5.05 Å is used to set the density of the 18-molecules water cluster to 1 kg/L.

### Machine-Learned Total Energy Function

The total energy function *E*_tot_^*X*^(ν), where ν is any configuration of the system *X*, is ML from a set of DFT calculations. For the latter, the Perdew–Burke–Ernzerhof (PBE) exchange correlation functional^20^ is used here in combination with pseudopotentials of the SSSP PBE efficiency 1.1.2 library for ionic cores.^21^ The system is placed in a 16 Å × 16 Å × 16 Å box, and the electrostatic potential of periodic images is removed by the parabolic electrostatic correction of Dabo et al.^22^ implemented in Environ.

The machine learning architecture is the E(3)-equivariant graph neural network (NN) Nequip of Batzner et al.^23^ Two NN models (NN-1 and NN-2) are used, with respective cutoff radii of the convolution filter of 5 and 6 Å, and respective numbers of interaction blocks of 2 and 3. The models are similar for all other parameters: a maximum rotation order of 2; a multiplicity of the features of 8; no odd parity; a “default” radial NN comprising 8 basis radial functions, 3 layers, and 64 hidden neurons. Each NN model is trained over 100 epochs with the Adam optimizer, putting equal weights on forces and the total energy per atom in the cost function. The optimized model is then exported and used in LAMMPS^24^ to perform MD simulations.

**Figure 2:**
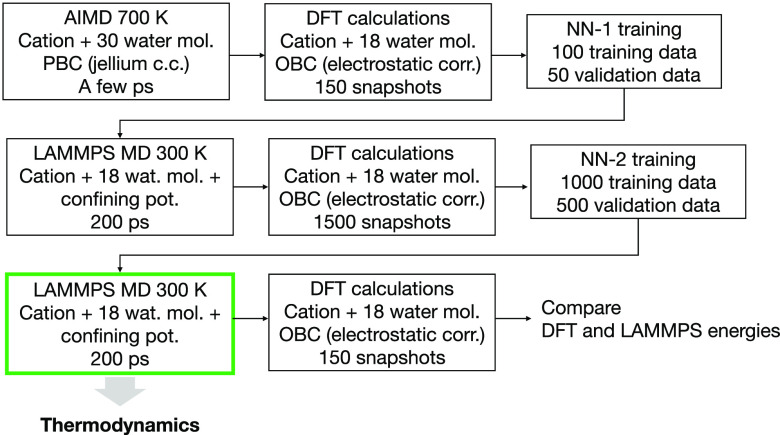
Computational workflow to produce a MD trajectory converged with respect to cross-validation DFT calculations.

The overall ML workflow (Figure 2) consists of 3 main stages:

- Stage 1 (first row): an initial FPMD trajectory is produced in PBC with Quantum ESPRESSO. A high temperature (700 K) is used to widen the sampling of bond length distributions and thus improve the robustness of the subsequent NN. From this trajectory, 150 random snapshots of the inner water cluster are extracted, and recalculated with DFT in open-boundary conditions (OBC) using the parabolic correction.^22^ This data set is then used to train the first model NN-1.
- Stage 2 (second row): a MD trajectory of 200 ps is produced with LAMMPS at a temperature of 300 K using the NN-1 model. From this trajectory, 1500 random snapshots are recalculated with DFT in OBC, and then used to train the more accurate NN-2 model.
- Stage 3 (third row): a new MD trajectory of 200 ps is produced using the NN-2 model. A new selection of 150 random snapshots is recalculated with DFT. If the LAMMPS energies compare well with DFT energies, the workflow is considered converged, and the relevant thermodynamic quantities are extracted from the last MD simulation.

## Results

The methodology has been applied to 5 alkaline (Li^+^ to Cs^+^) and 5 alkaline-earth (Be^2+^ to Ba^2+^) cations.

For *n* = 18 and *r*_c_ = 5.05 Å, we find *P*_0_(*n*) ≈ 0.24, and the *P*_*i*_(*n*) values are reported in Table 1. According to eq 1, the *P*_0_(*n*)/*P*_*i*_(*n*) ratio then contributes a maximum free energy shift of −0.02 eV. Although approximate, this result indicates that given the small size of the solute, the *P*_0_(*n*) and *P*_*i*_(*n*) distributions largely overlap, and for the *n* and *r*_c_ values chosen here, the corresponding free energy contribution is small. In the following, we thus approximate Δ*G*_solv_^ion^ ≈ Δ*G*_*i*_(*n*).

**Table 1:** *P*_*i*_(*n*) Values for Alkaline(-Earth) Ions with *n* = 18 and *r*_c_ = 5.05 Å

*i*	Li^+^	Na^+^	K^+^	Rb^+^	Cs^+^	Be^2+^	Mg^2+^	Ca^2+^	Sr^2+^	Ba^2+^
*P*_*i*_(*n*)	0.16	0.25	0.19	0.24	0.24	0.19	0.12	0.16	0.10	0.22

For Δ*G*_*i*_(*n*), the calculation workflow has been converged within a chemical accuracy of 0.04 eV, by checking that (i) the mean LAMMPS and DFT absolute energy difference is less than 0.04 eV over the last cross-validation set, and (ii) the mean total energy ⟨*E*_tot_^*X*^⟩ of the last MD simulation is converged within 0.04 eV.

The calculated solvation energies are reported in Table 2. Our results are in general good agreement with the previously calculated values of Xi et al. and experimental values. For alkaline-earth cations, the agreement with experiment is also improved, leaving only Be^2+^ and Mg^2+^ slightly above the reported experimental range.

**Table 2:** Calculated and Experimental^25−31^ Absolute Values of the Free Energies of Solvation^a^

cation	calc. (Xi et al.)	calc. (this work)	exp.
Li^+^	5.46	5.05	4.92–5.57
Na^+^	4.31	4.10	3.78–4.47
K^+^	3.42	3.31	3.06–3.73
Rb^+^	3.03	3.15	2.85–3.49
Cs^+^	2.90	2.86	2.59–3.26
Be^2+^	26.82	25.71	24.82–25.07
Mg^2+^	20.45	19.60	18.97–19.21
Ca^2+^	16.74	16.34	15.60–16.59
Sr^2+^	15.34	14.80	14.30–15.08
Ba^2+^	14.00	13.40	12.95–13.74
MRE	4.3%	1.8%	

a The MRE is the mean relative error of the calculated value vs the average experimental value. The same standard state correction is applied as in eq 20 of ref (14).

## Conclusions

The hybrid solvation method of Xi et al. has been re-expressed in the QCT framework and extended/modified to run longer MD simulations at a fraction of the computational cost. The extension consists mainly in using a FP-based ML force field in the MD, leveraging the data-efficient O(3)-equivariant NN Nequip architecture. Another modification is to treat the outer solvation environment analytically, in contrast with the implicit continuum medium of the original approach. The extended approach is tested by running 200 ps MD simulations on alkaline(-earth) cations and converging the mean energy within chemical accuracy (0.04 eV). The calculated solvation free energies are in good agreement with previously calculated values and experimental values.

## Data Availability

MD trajectories, energies and forces used to obtain the solvation energies of Table 2 are available on Materials Cloud (DOI: 10.24435/materialscloud:a0-jh).
